# Supplementary material for: Rapid evolution of Klebsiella pneumoniae biofilms in vitro delineates adaptive changes selected during infection
Source: Nat Commun. 2026 Apr 10;17:3454. doi: 10.1038/s41467-026-71505-w (PMC13076756; doi:10.1038/s41467-026-71505-w)
Supplement: Supplementary file 1 — Supplementary Information [file 41467_2026_71505_MOESM1_ESM.pdf]

Supplementary information

"Rapid evolution of *Klebsiella pneumoniae* biofilms *in vitro* delineates adaptive changes selected during infection"

Greta Zaborskytė[1]*, Patrícia Coelho[1,2], Marie Wrande[1], Linus Sandegren[1,2]

[1]Department of Medical Biochemistry and Microbiology, Uppsala University, Uppsala, Sweden.
[2]Uppsala Antibiotic Center, Uppsala University, Uppsala, Sweden.
*Current address: Ineos Oxford Institute for Antimicrobial Research, Department of Chemistry, University of Oxford, Oxford, UK.

Corresponding author: linus.sandegren@imbim.uu.se

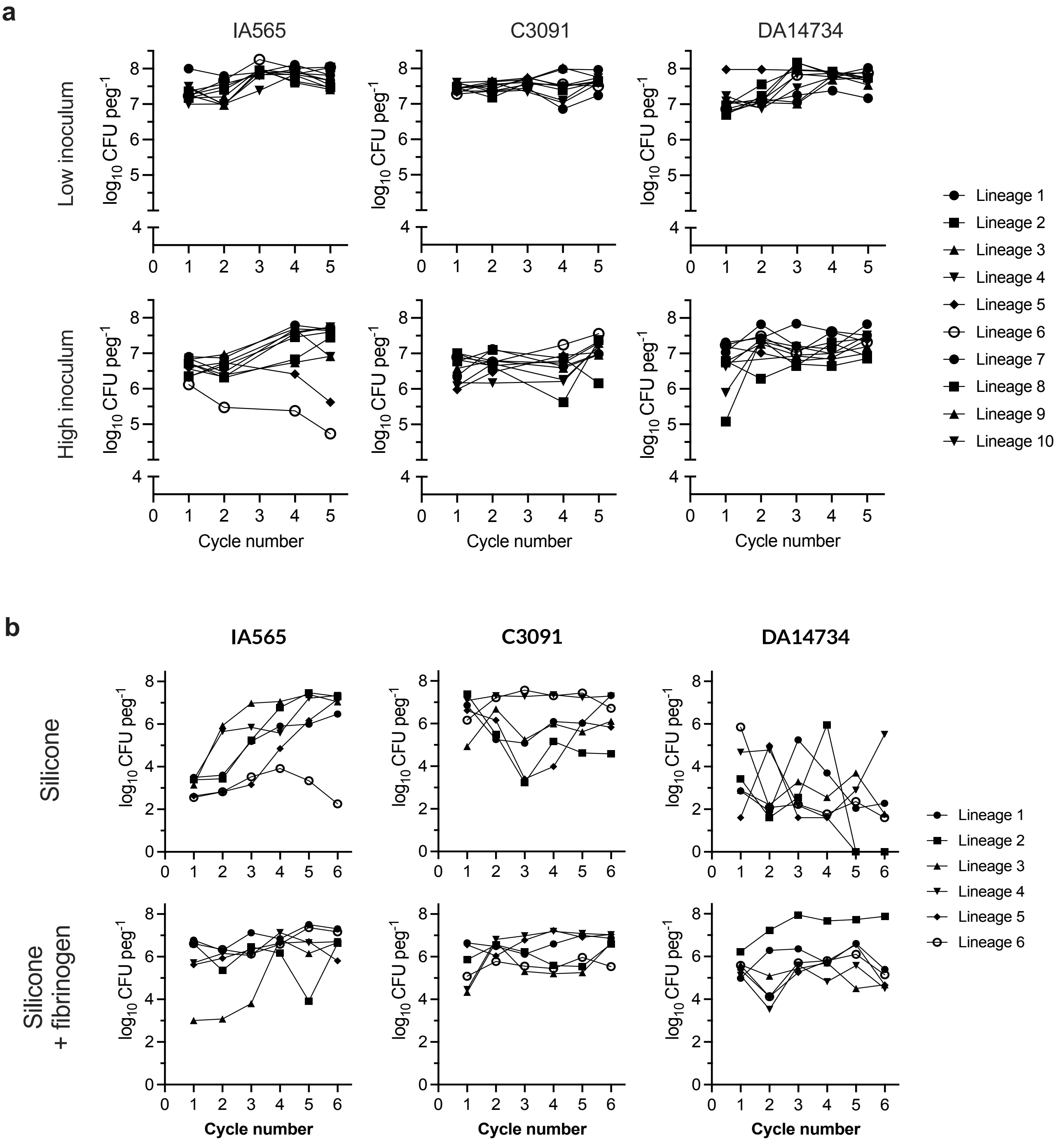

Supplementary Fig. 1 | Population size changes during serial passaging on **a,** uncoated pegs and **b,** silicone-coated pegs with (bottom) and without (top) fibrinogen during serial passaging in BHI medium. Source data are provided as a Source Data file.

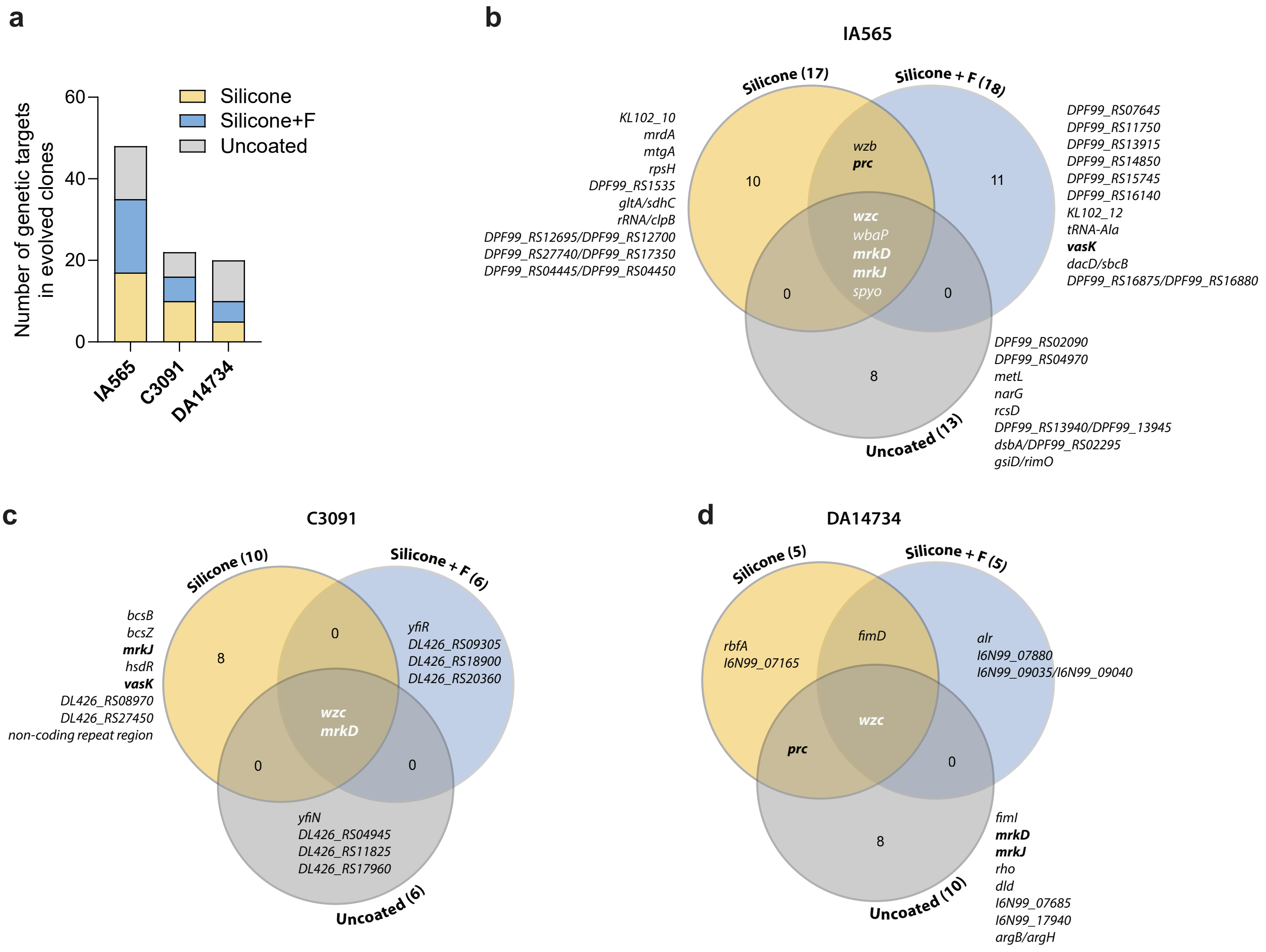

Supplementary Fig. 2 | Mutational targets in evolved clones. a Overall number of mutated targets (genes with nonsynonymous mutations and intergenic regions) after selection on different surfaces for IA565, C3091, and DA14734 strains. **b, c, d** Venn diagrams showing the overlap of mutated genetic targets. Genes marked in bold were mutated in more than one strain. Source data are provided as a Source Data file.

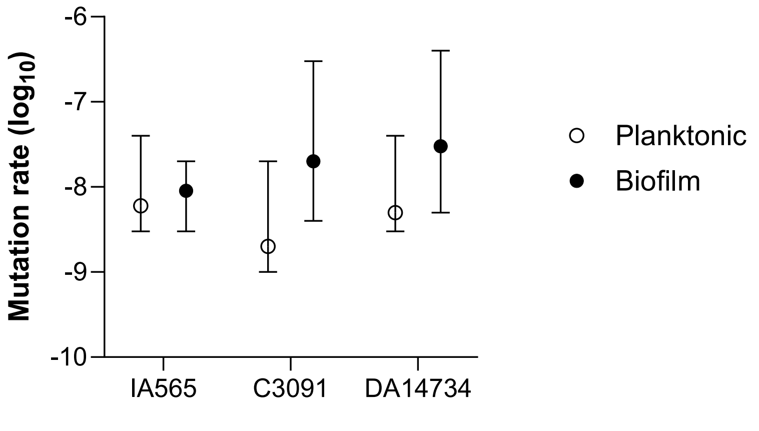

Supplementary Fig. 3 | Mutation rates in planktonic and biofilm states. Mutation rates to rifampicin resistance were measured using fluctuation test and the median method by Lea & Caulson with 24 independent cultures/biofilms per strain. Data are shown as median with 95% CI. Source data are provided as a Source Data file.

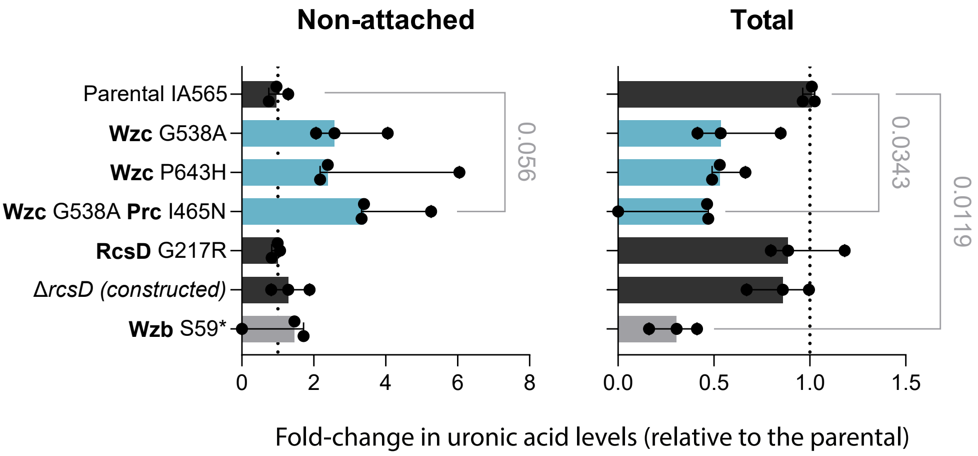

Supplementary Fig. 4 | Polysaccharide quantification in the IA565 genetic background by uronic acid assay. Non-attached and total extracellular polysaccharide content was measured from the parental and mutant strains. Median values from three biological replicates with 95% CI are shown. Strains with parental colony morphotypes are in dark grey, hypermucoid in light blue, and translucent in light grey. Statistical significance was determined by Kruskal-Wallis test followed by Dunn's multiple comparison test. Source data are provided as a Source Data file.

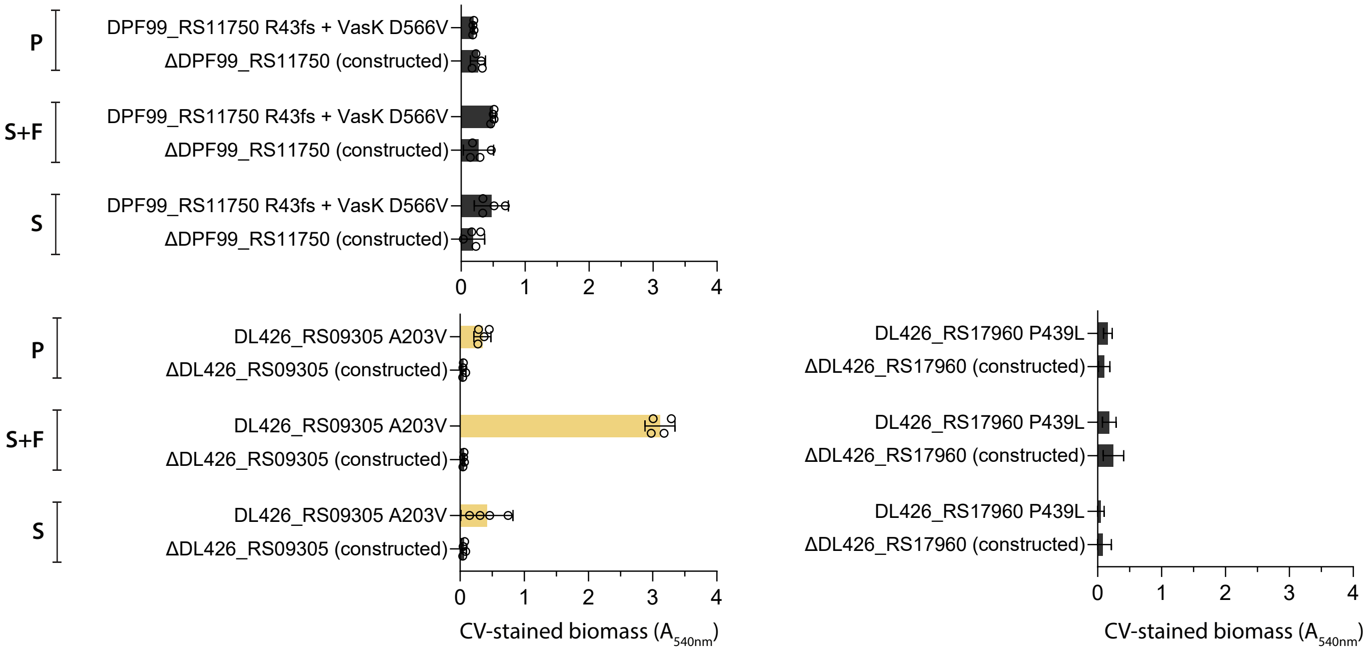

Supplementary Fig. 5 | Comparison of biofilm capacity in evolved mutants and corresponding constructed deletion strains. Means with 95% CI of CV-stained biofilm biomass on silicone pegs, silicone + fibrinogen pegs, and in polystyrene wells after 48 h growth (n=4 biological replicates). Source data are provided as a Source Data file.

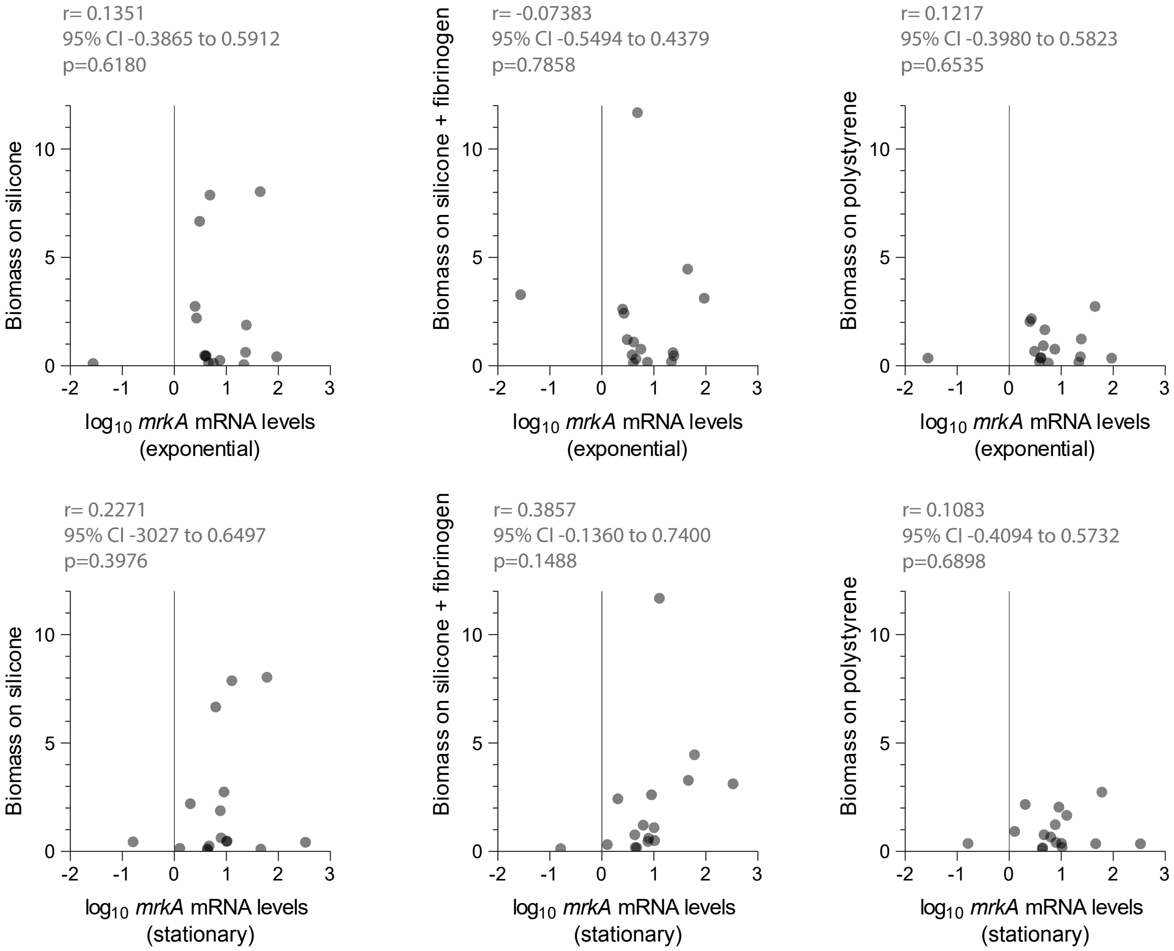

Supplementary Fig. 6 | Interconnection between biofilm formation on different surfaces and expression of *mrkA* at exponential (top panel) and stationary growth phase (bottom panel) for individual mutants (n=16). Each dot represents mean values from biofilm formation assays and measurements of *mrkA* mRNA levels. Pearson correlation coefficients (r) with 95% CI and two-sided p-values are shown. Source data are provided as a Source Data file.

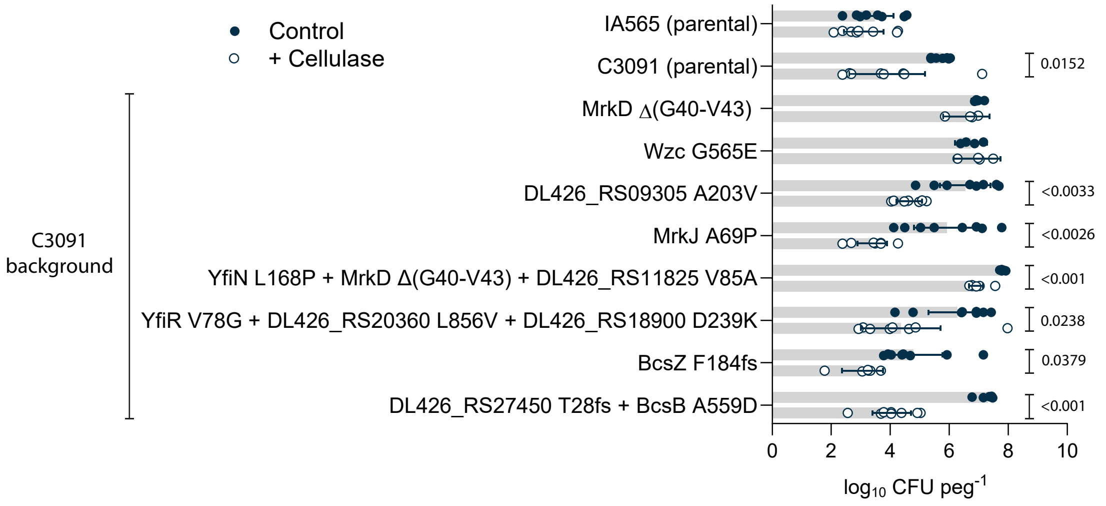

Supplementary Fig. 7 | Biofilm growth on silicone-coated pegs (24 h) in the presence of cellulase enzyme. Means from n=8 biological replicates with 95% CI are shown. Statistical significance was assessed by a paired two-sided Student's t-test between control and treated biofilms for each strain; p-values exceeding significance (p<0.05) are shown. Source data are provided as a Source Data file.

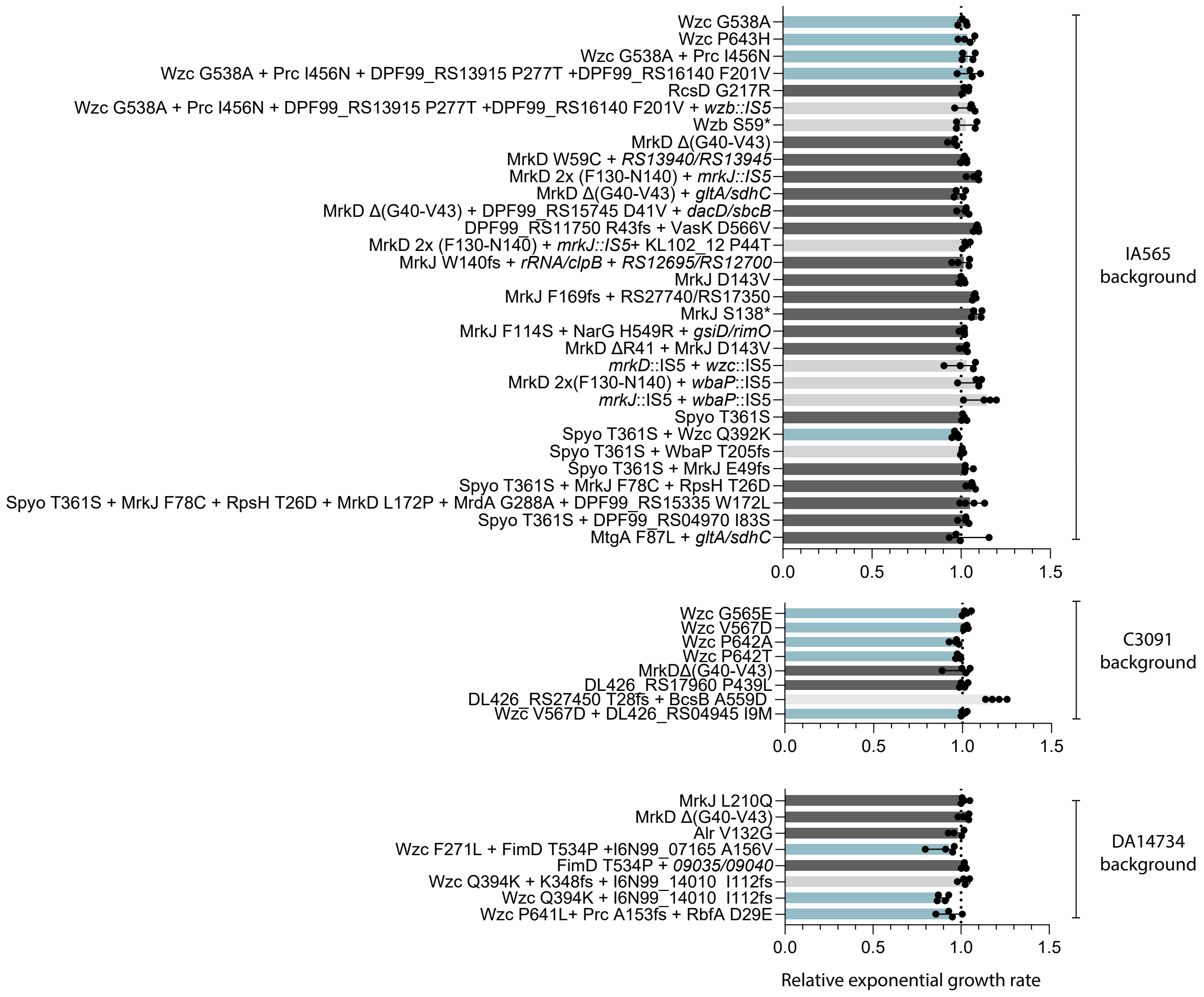

Supplementary Fig. 8 | Exponential growth rates in biofilm-evolved mutants during planktonic growth in BHI relative to the parental strain. Bars show means of n=5 biological replicates with 95% CI. Source data are provided as a Source Data file.
