# Supplementary material for: Rapid evolution of Klebsiella pneumoniae biofilms in vitro delineates adaptive changes selected during infection
Source: Nat Commun. 2026 Apr 10;17:3454. doi: 10.1038/s41467-026-71505-w (PMC13076756; doi:10.1038/s41467-026-71505-w)
Supplement: Supplementary file 2 — Description of Additional Supplementary Files [file 41467_2026_71505_MOESM2_ESM.pdf]

Description of Additional Supplementary Files:

Supplementary Data 1: Trajectories and frequencies of clones in all silicone/silicone+fibrinogen lineages.

Supplementary Data 2: Tables with all mutations in each clone and population for the three parental strains.

Supplementary Data 3: Primer sequences used for constructing in-frame deletions, screening for deletions, and qPCR.
