# Supplementary material for: Rapid evolution of Klebsiella pneumoniae biofilms in vitro delineates adaptive changes selected during infection
Source: Nat Commun. 2026 Apr 10;17:3454. doi: 10.1038/s41467-026-71505-w (PMC13076756; doi:10.1038/s41467-026-71505-w)

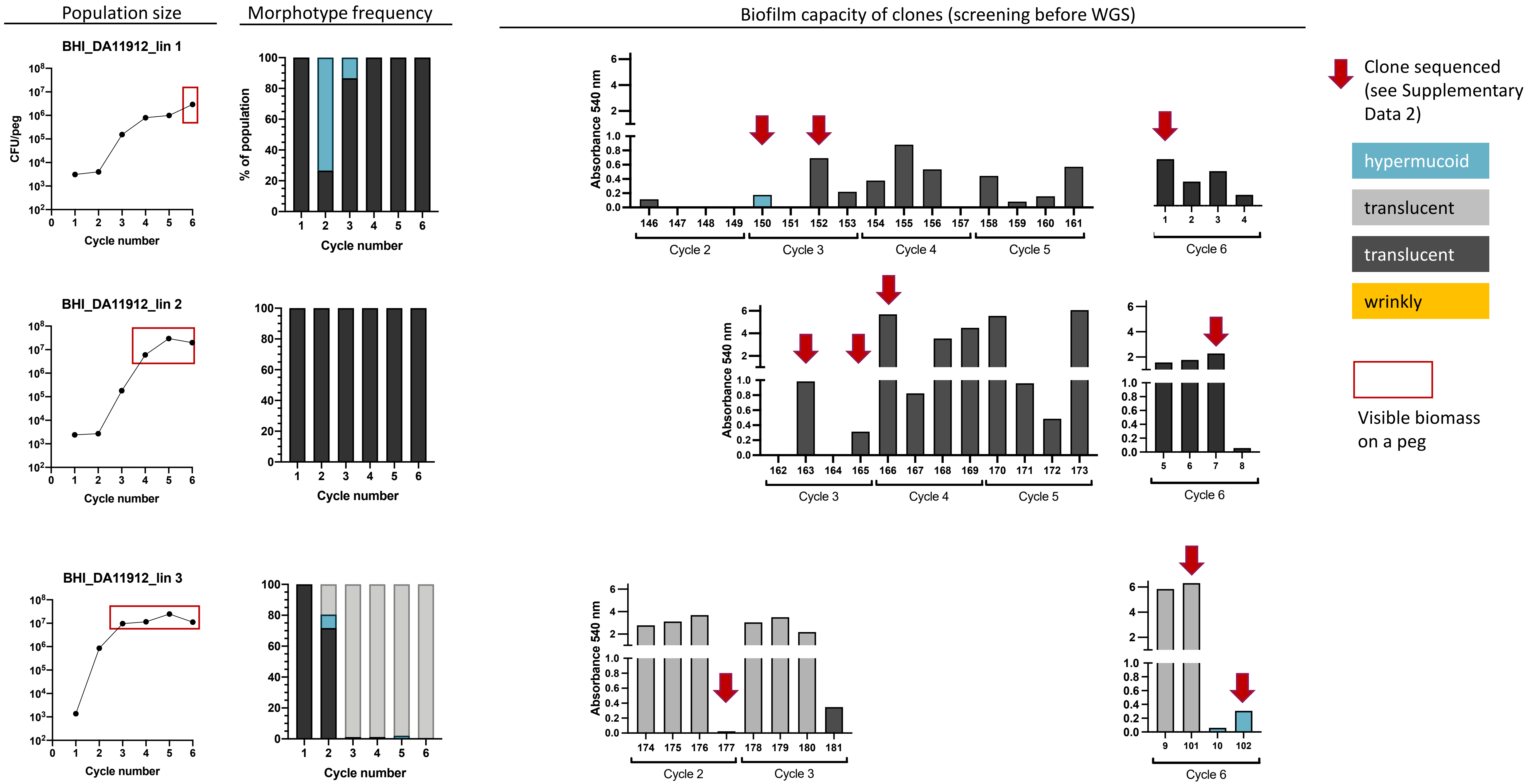

Population size
Morphotype frequency
Biofilm capacity of clones (screening before WGS)
BHI_DA11912_lin 1
BHI_DA11912_lin 2
BHI_DA11912_lin 3
CFU/peg
Cycle number
% of population
Absorbance 540 nm
Cycle 2
Cycle 3
Cycle 4
Cycle 5
Cycle 6
Clone sequenced (see Supplementary Data 2)
hypermucoid
translucent
translucent
wrinkly
Visible biomass on a peg

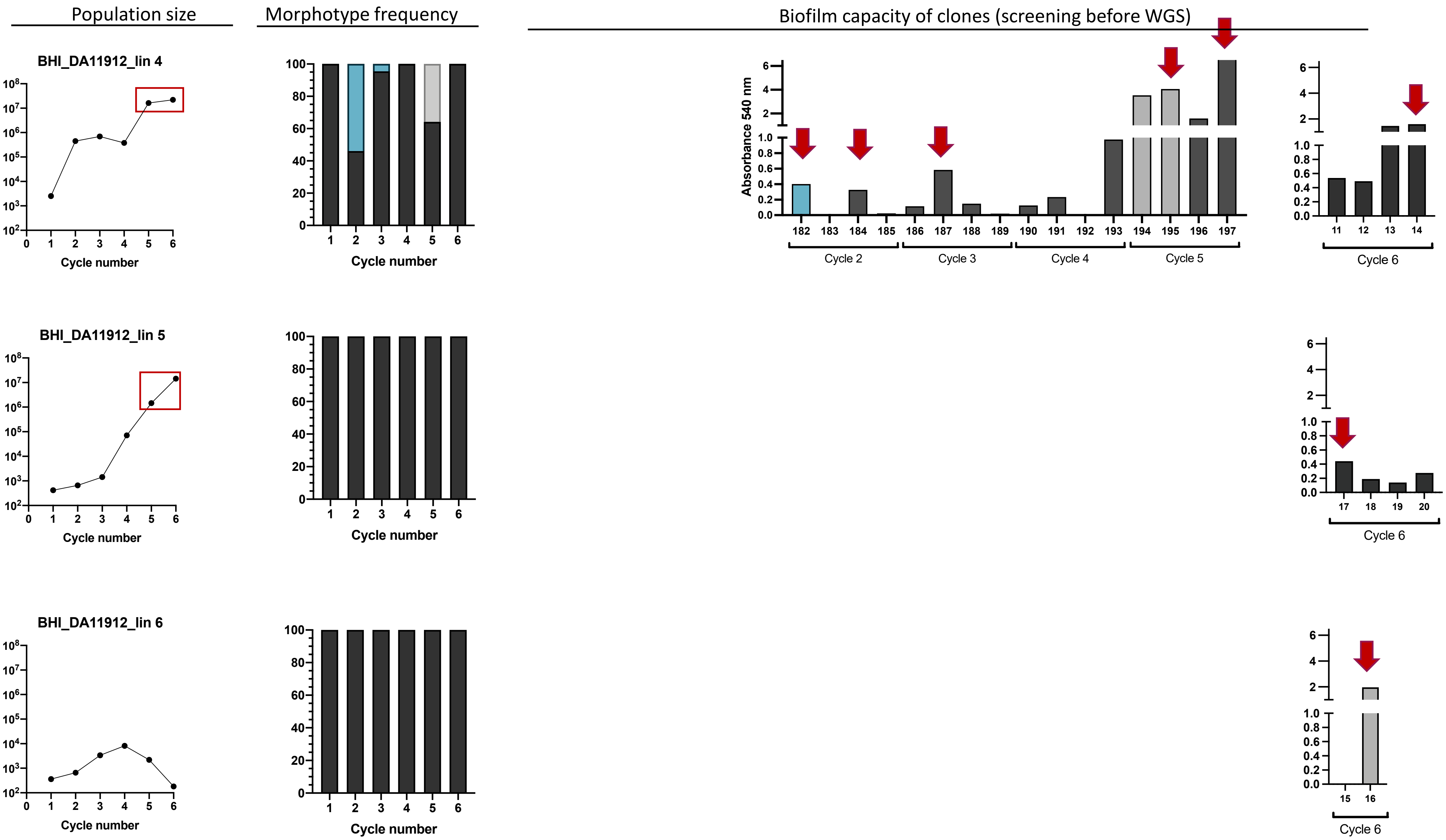
Population size
Morphotype frequency
Biofilm capacity of clones (screening before WGS)
BHI_DA11912_lin 4
BHI_DA11912_lin 5
BHI_DA11912_lin 6
Cycle number
Absorbance 540 nm
182
183
184
185
186
187
188
189
190
191
192
193
194
195
196
197
Cycle 2
Cycle 3
Cycle 4
Cycle 5
11
12
13
14
Cycle 6
17
18
19
20
Cycle 6
15
16
Cycle 6

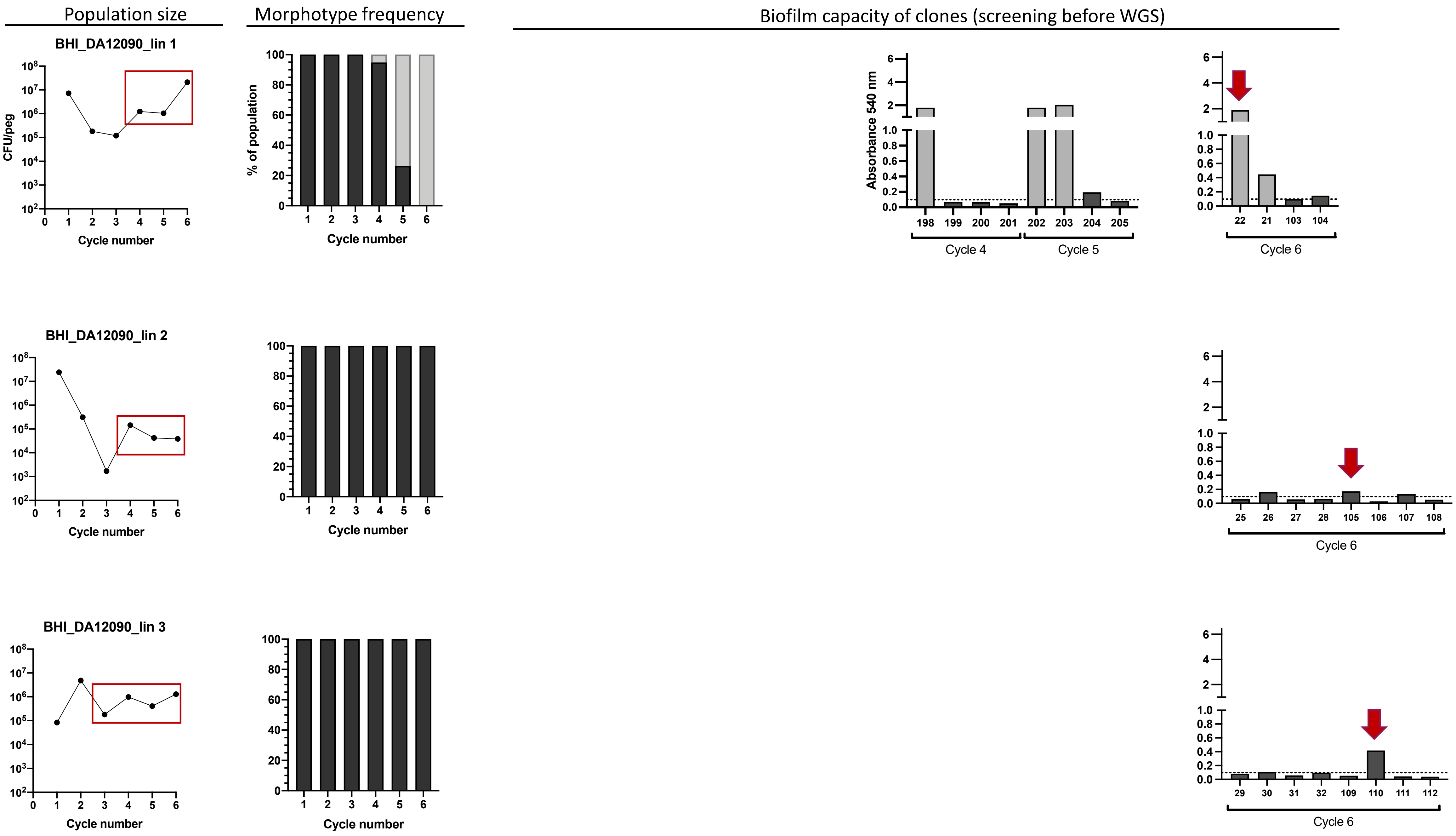

Population size
Morphotype frequency
Biofilm capacity of clones (screening before WGS)
BHI_DA12090_lin 1
BHI_DA12090_lin 2
BHI_DA12090_lin 3
CFU/peg
% of population
Absorbance 540 nm
Cycle number
198
199
200
201
202
203
204
205
Cycle 4
Cycle 5
22
21
103
104
Cycle 6
25
26
27
28
105
106
107
108
Cycle 6
29
30
31
32
109
110
111
112
Cycle 6

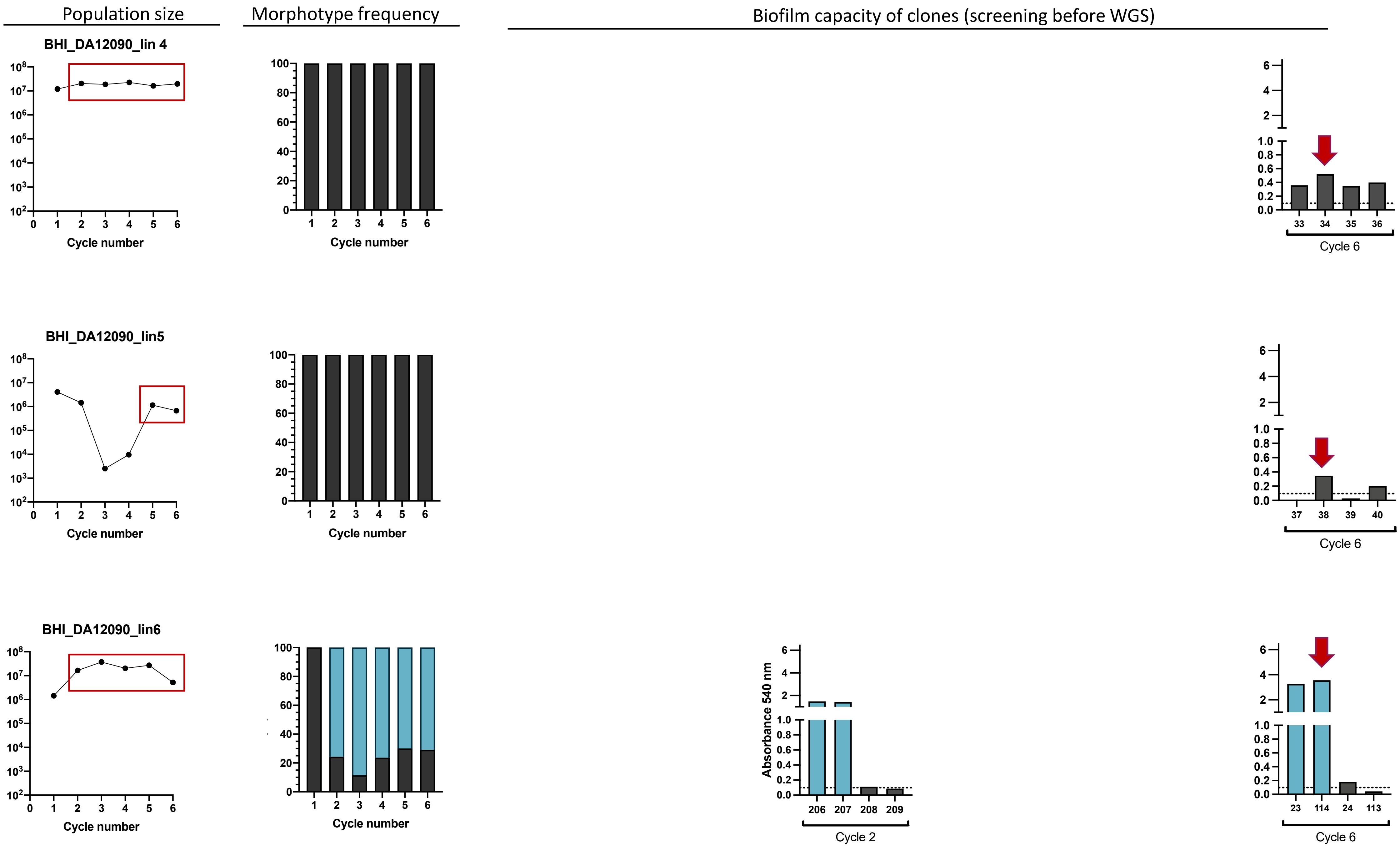

Population size
Morphotype frequency
Biofilm capacity of clones (screening before WGS)
BHI_DA12090_lin 4
BHI_DA12090_lin5
BHI_DA12090_lin6
Cycle number
Absorbance 540 nm
Cycle 2
Cycle 6

Population size | Morphotype frequency | Biofilm capacity of clones (screening before WGS)

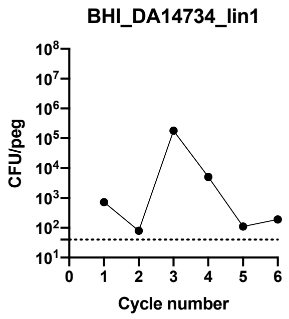

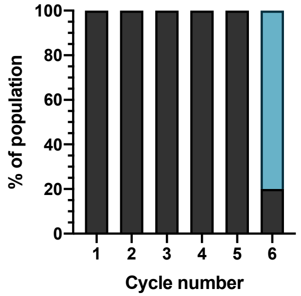

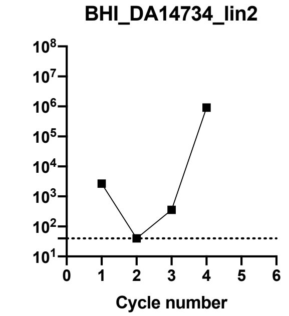

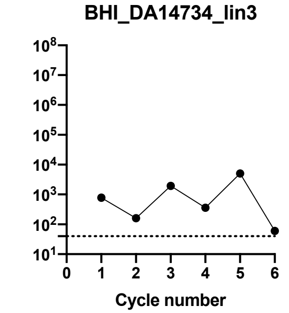

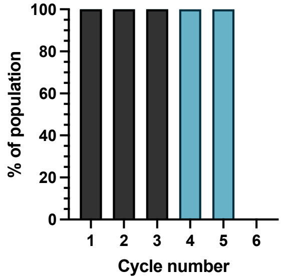

Population size

Morphotype frequency

Biofilm capacity of clones (screening before WGS)

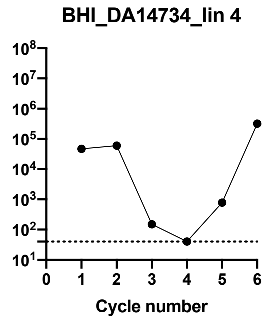

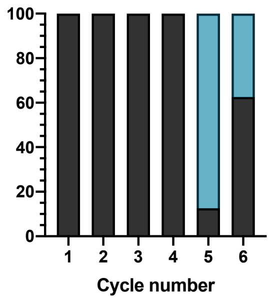

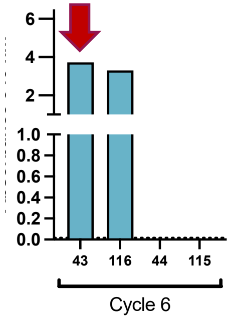

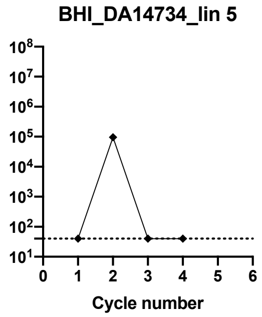

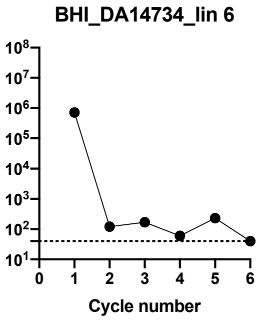

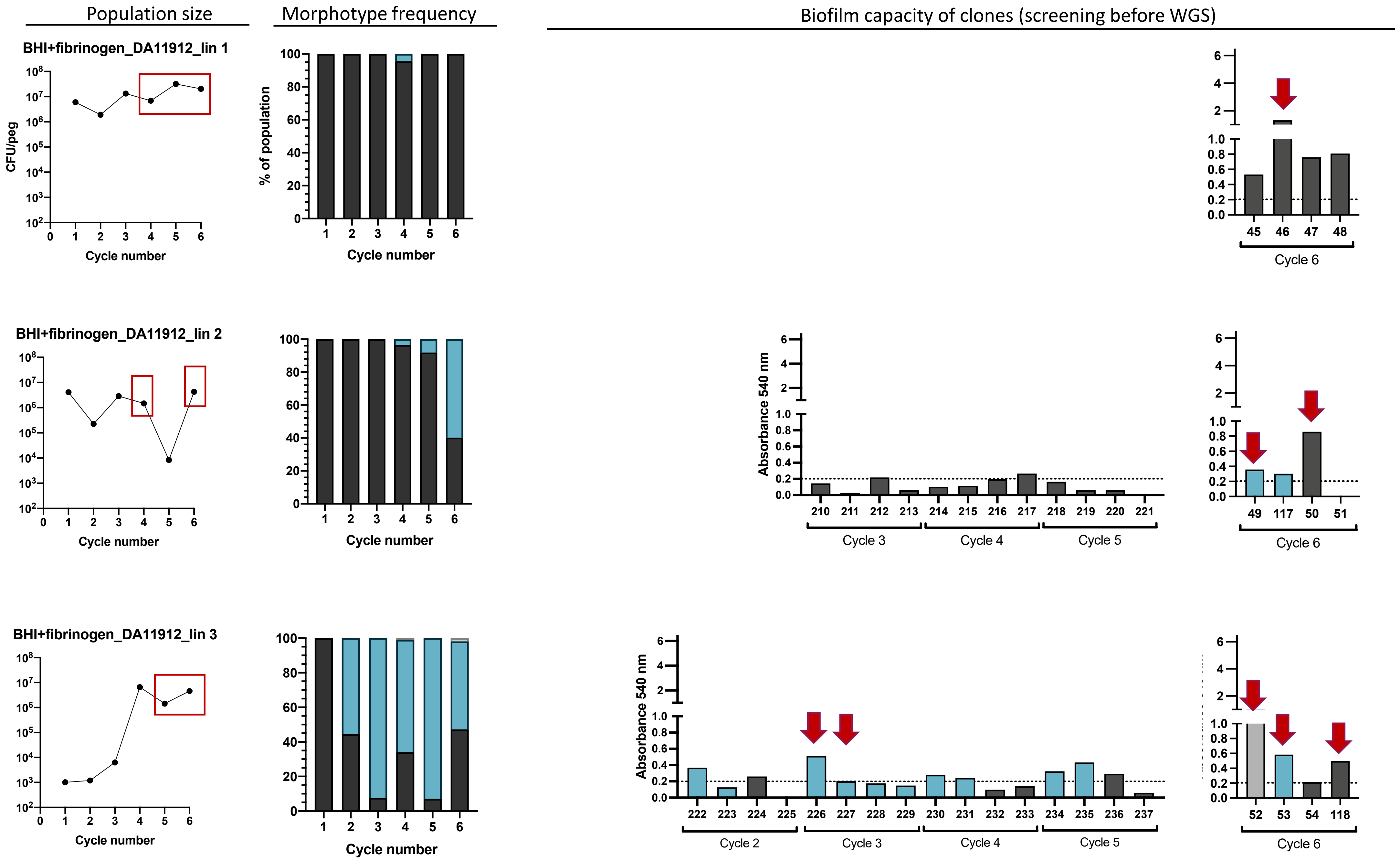

Population size
Morphotype frequency
Biofilm capacity of clones (screening before WGS)
BHI+fibrinogen_DA11912_lin 1
BHI+fibrinogen_DA11912_lin 2
BHI+fibrinogen_DA11912_lin 3
CFU/peg
% of population
Absorbance 540 nm
Cycle number
Cycle 2
Cycle 3
Cycle 4
Cycle 5
Cycle 6

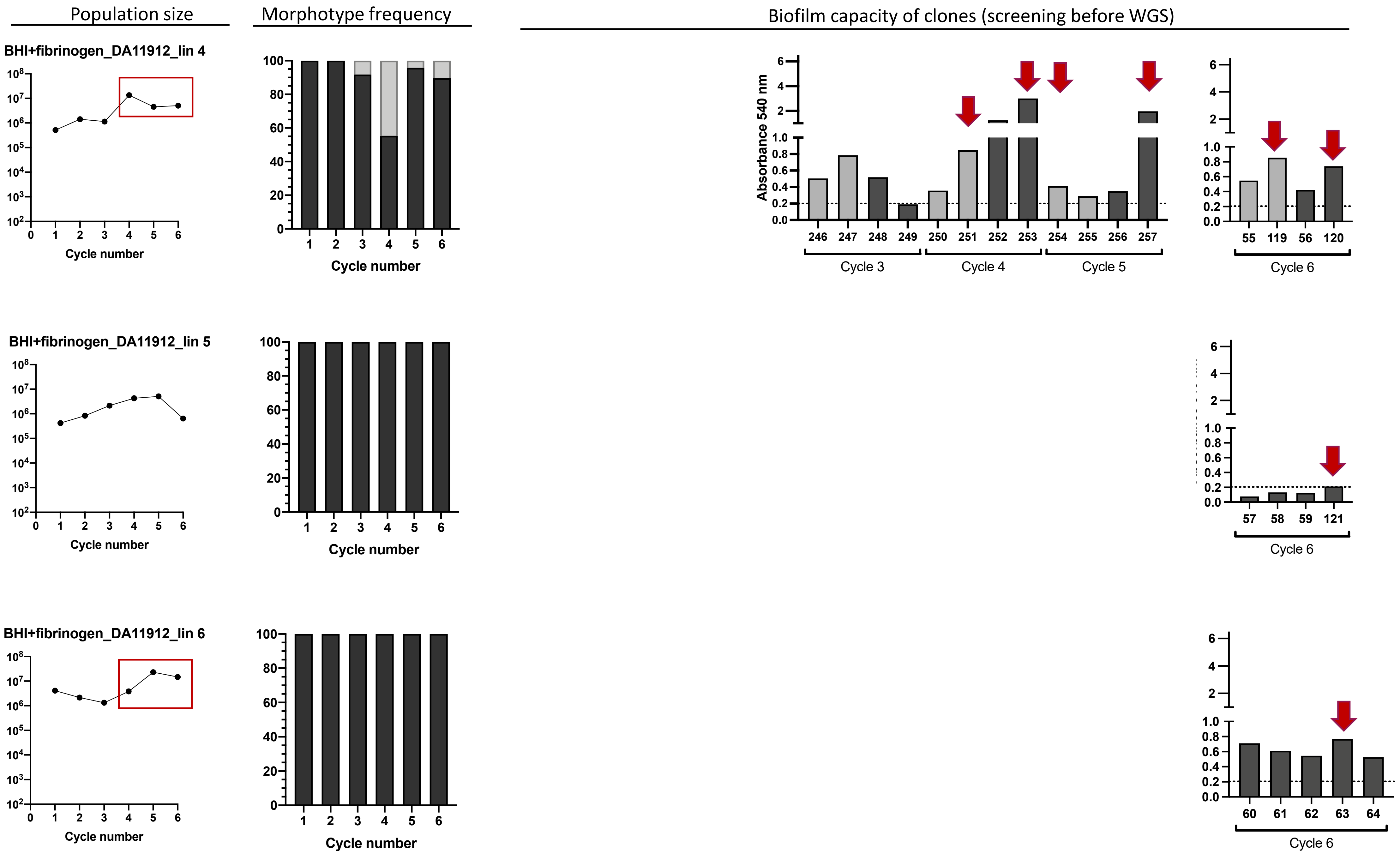
Population size
Morphotype frequency
Biofilm capacity of clones (screening before WGS)
BHI+fibrinogen_DA11912_lin 4
BHI+fibrinogen_DA11912_lin 5
BHI+fibrinogen_DA11912_lin 6
Cycle number
Absorbance 540 nm
246 247 248 249 250 251 252 253 254 255 256 257
Cycle 3
Cycle 4
Cycle 5
55 119 56 120
Cycle 6
57 58 59 121
Cycle 6
60 61 62 63 64
Cycle 6

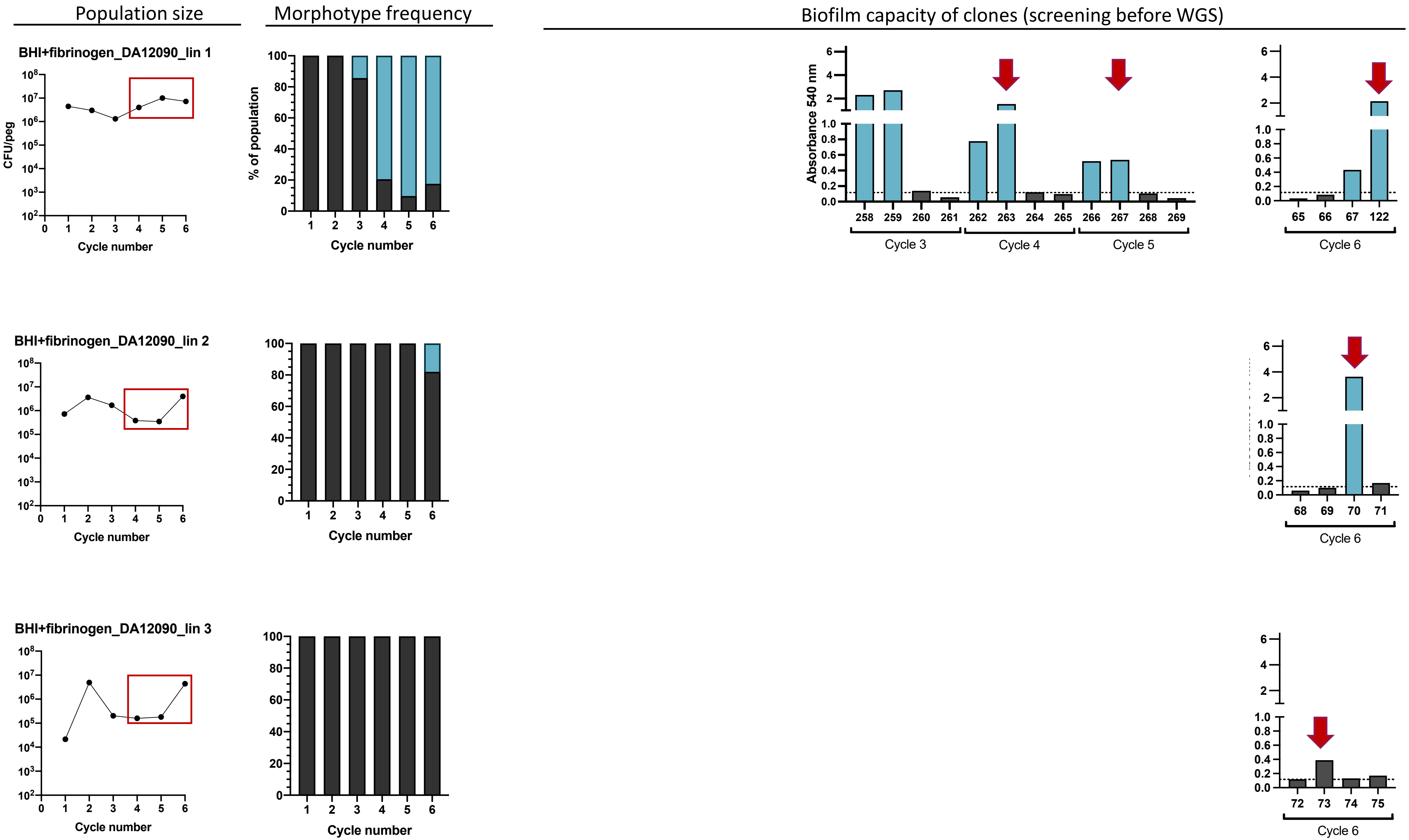
Population size
Morphotype frequency
Biofilm capacity of clones (screening before WGS)
BHI+fibrinogen_DA12090_lin 1
BHI+fibrinogen_DA12090_lin 2
BHI+fibrinogen_DA12090_lin 3
CFU/peg
10^2
10^3
10^4
10^5
10^6
10^7
10^8
Cycle number
% of population
0
20
40
60
80
100
Absorbance 540 nm
0.0
0.2
0.4
0.6
0.8
1.0
2
4
6
258
259
260
261
262
263
264
265
266
267
268
269
Cycle 3
Cycle 4
Cycle 5
65
66
67
122
68
69
70
71
72
73
74
75
Cycle 6

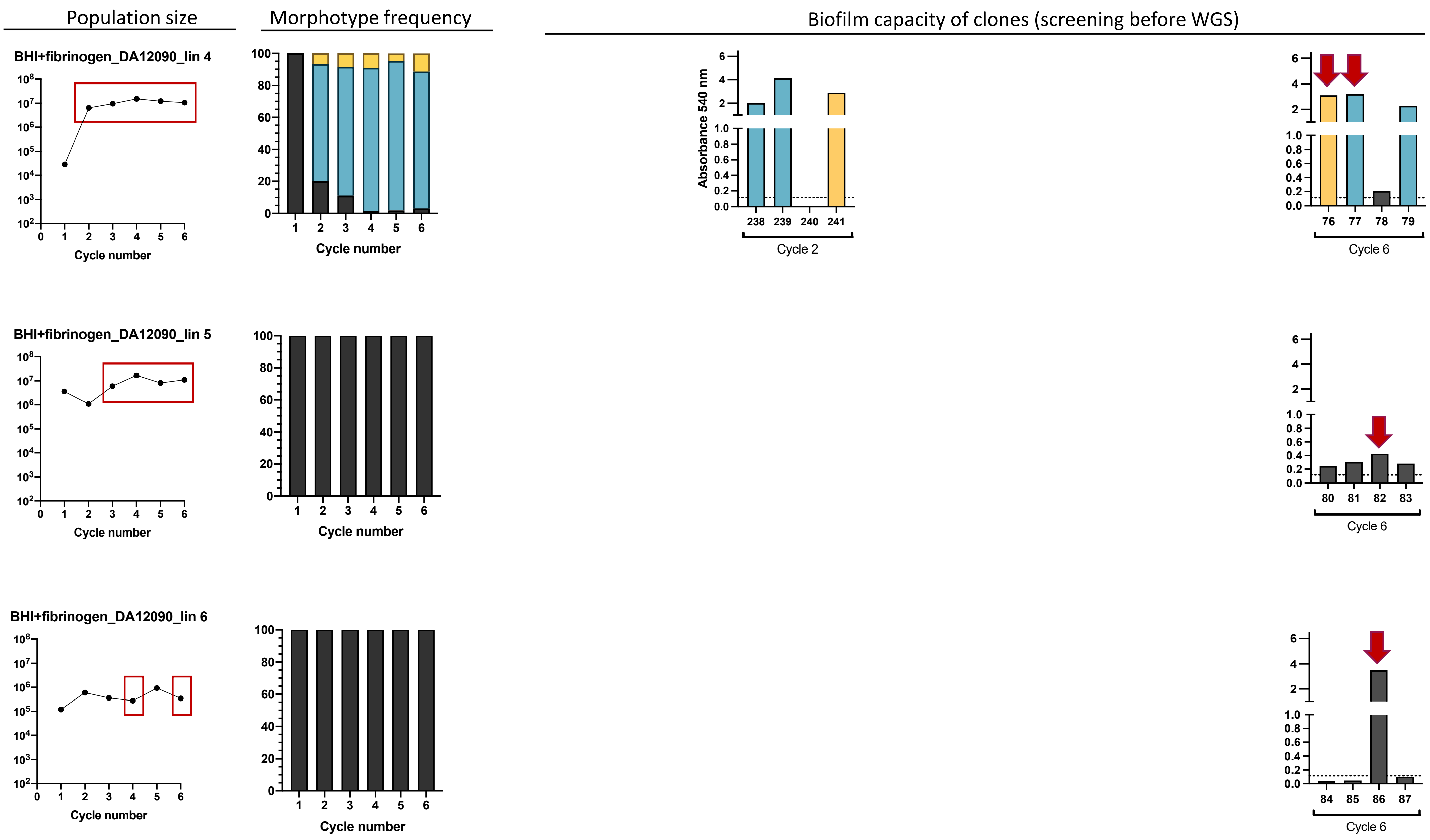

Population size
Morphotype frequency
Biofilm capacity of clones (screening before WGS)
BHI+fibrinogen_DA12090_lin 4
BHI+fibrinogen_DA12090_lin 5
BHI+fibrinogen_DA12090_lin 6
Cycle number
Absorbance 540 nm
238
239
240
241
Cycle 2
76
77
78
79
80
81
82
83
84
85
86
87
Cycle 6

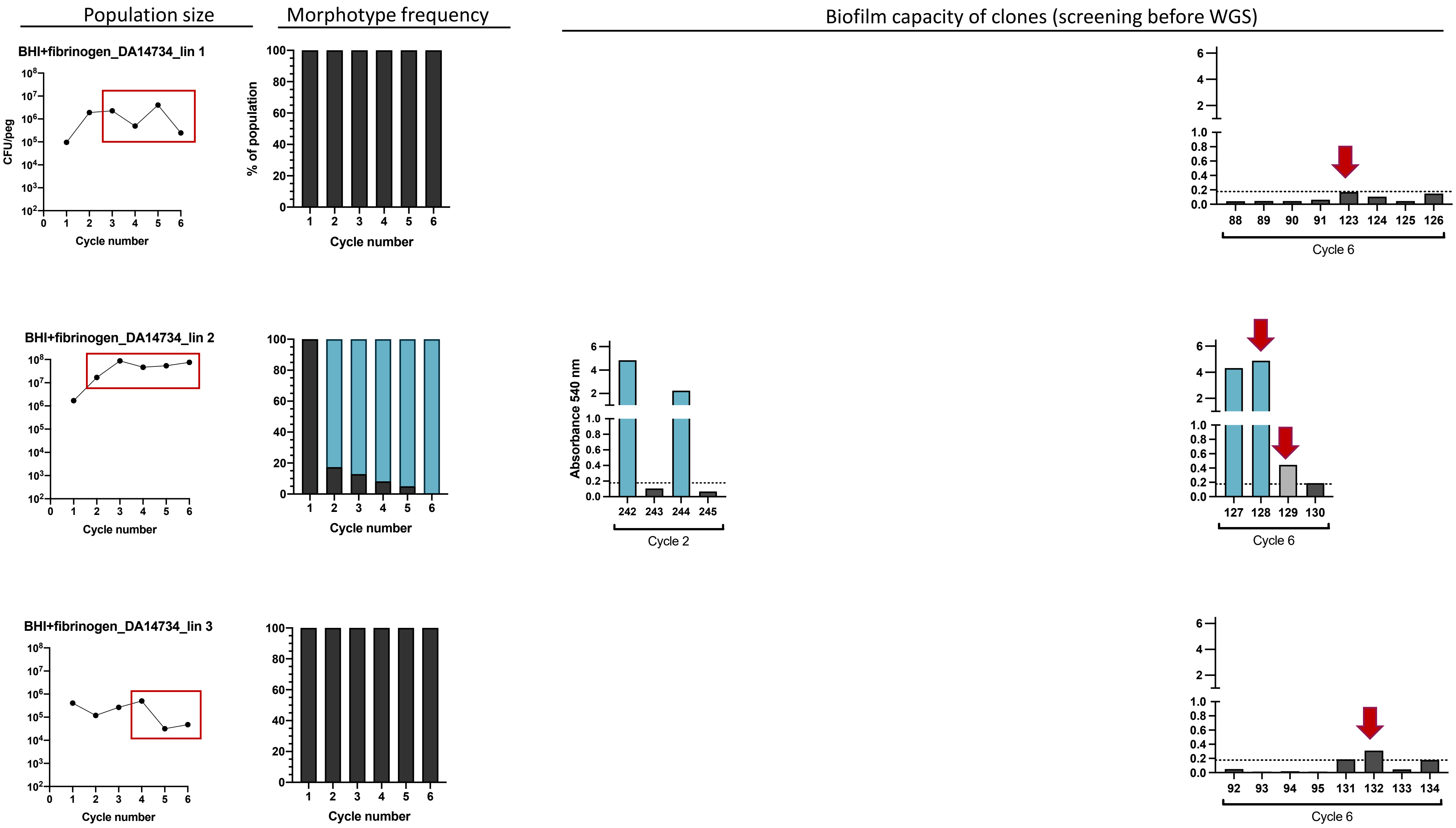
Population size
Morphotype frequency
Biofilm capacity of clones (screening before WGS)
BHI+fibrinogen_DA14734_lin 1
BHI+fibrinogen_DA14734_lin 2
BHI+fibrinogen_DA14734_lin 3
CFU/peg
Cycle number
% of population
Absorbance 540 nm
Cycle 6
Cycle 2
88 89 90 91 123 124 125 126
242 243 244 245
127 128 129 130
92 93 94 95 131 132 133 134

Population size | Morphotype frequency | Biofilm capacity of clones (screening before WGS)

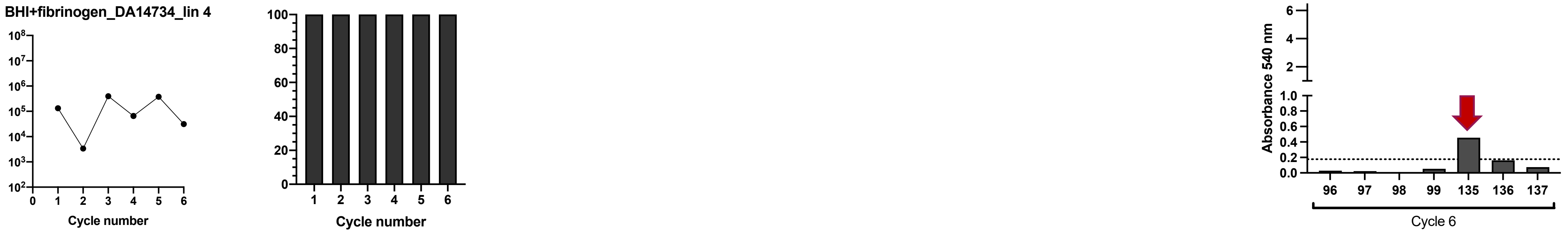

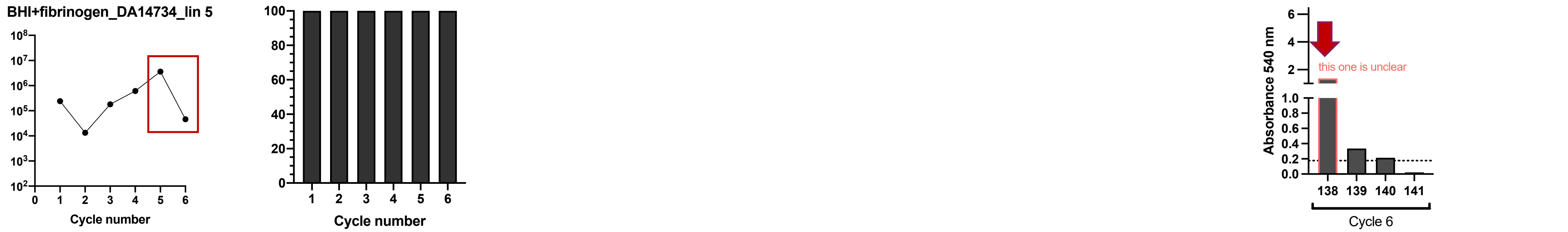

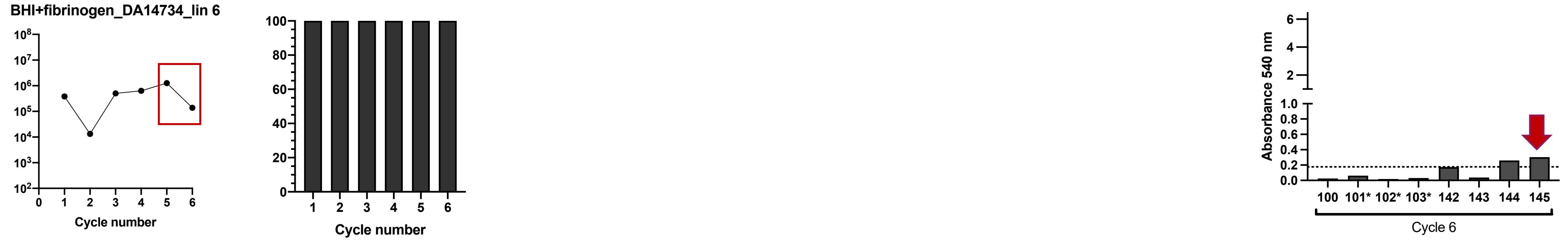

Supplement: Supplementary file 3 — Supplementary Data 1 [file 41467_2026_71505_MOESM3_ESM.pdf]
