# Supplementary material for: Rapid evolution of Klebsiella pneumoniae biofilms in vitro delineates adaptive changes selected during infection
Source: Nat Commun. 2026 Apr 10;17:3454. doi: 10.1038/s41467-026-71505-w (PMC13076756; doi:10.1038/s41467-026-71505-w)
Supplement: Supplementary file 6 — Reporting Summary [file 41467_2026_71505_MOESM6_ESM.pdf]

nature portfolio

Corresponding author(s): Linus Sandegren

Last updated by author(s): Oct 9, 2025

# Reporting Summary

Nature Portfolio wishes to improve the reproducibility of the work that we publish. This form provides structure for consistency and transparency in reporting. For further information on Nature Portfolio policies, see our Editorial Policies and the Editorial Policy Checklist.

## Statistics

For all statistical analyses, confirm that the following items are present in the figure legend, table legend, main text, or Methods section.

| n/a | Confirmed | |
|---|---|---|
| ☐ | ☒ | The exact sample size (*n*) for each experimental group/condition, given as a discrete number and unit of measurement |
| ☐ | ☒ | A statement on whether measurements were taken from distinct samples or whether the same sample was measured repeatedly |
| ☐ | ☒ | The statistical test(s) used AND whether they are one- or two-sided<br>*Only common tests should be described solely by name; describe more complex techniques in the Methods section.* |
| ☒ | ☐ | A description of all covariates tested |
| ☐ | ☒ | A description of any assumptions or corrections, such as tests of normality and adjustment for multiple comparisons |
| ☐ | ☒ | A full description of the statistical parameters including central tendency (e.g. means) or other basic estimates (e.g. regression coefficient) AND variation (e.g. standard deviation) or associated estimates of uncertainty (e.g. confidence intervals) |
| ☐ | ☒ | For null hypothesis testing, the test statistic (e.g. *F*, *t*, *r*) with confidence intervals, effect sizes, degrees of freedom and *P* value noted<br>*Give P values as exact values whenever suitable.* |
| ☒ | ☐ | For Bayesian analysis, information on the choice of priors and Markov chain Monte Carlo settings |
| ☒ | ☐ | For hierarchical and complex designs, identification of the appropriate level for tests and full reporting of outcomes |
| ☐ | ☒ | Estimates of effect sizes (e.g. Cohen's *d*, Pearson's *r*), indicating how they were calculated |

*Our web collection on statistics for biologists contains articles on many of the points above.*

## Software and code

Policy information about availability of computer code

| | |
|---|---|
| Data collection | No software was used for data collection. |
| Data analysis | Genomic analysis was performed using CLC Genomics Workbench v.25 (Qiagen). Statistical analyses were performed using GraphPad Prism version 9.2.0. for macOS (GraphPad Software, San Diego, California, USA). |

For manuscripts utilizing custom algorithms or software that are central to the research but not yet described in published literature, software must be made available to editors and reviewers. We strongly encourage code deposition in a community repository (e.g. GitHub). See the Nature Portfolio guidelines for submitting code & software for further information.

## Data

Policy information about availability of data

All manuscripts must include a data availability statement. This statement should provide the following information, where applicable:

- Accession codes, unique identifiers, or web links for publicly available datasets
- A description of any restrictions on data availability
- For clinical datasets or third party data, please ensure that the statement adheres to our policy

The complete genome sequences of IA565 (DA11912) and C3091 (DA12090) have been deposited under BioProject PRJNA473315 and PRJNA473316, respectively, and the outbreak index isolate under PRJNA857654. Sequence files for all mutants in this study have been deposited in the Short Read Archive (NCBI) under BioProject PRJNA1048869. Source data and Supplementary videos are provided with this paper and via figshare (https://doi.org/10.6084/m9.figshare.29816147). Biological material is available from the corresponding author upon reasonable request.

## Research involving human participants, their data, or biological material

Policy information about studies with human participants or human data. See also policy information about sex, gender (identity/presentation), and sexual orientation and race, ethnicity and racism.

| | |
|---|---|
| Reporting on sex and gender | The study is strictly involving bacterial strains and their evolution and included no human participand, data, or biological material. |
| Reporting on race, ethnicity, or other socially relevant groupings | The study is strictly involving bacterial strains and their evolution and included no human participand, data, or biological material. |
| Population characteristics | The study is strictly involving bacterial strains and their evolution and included no human participand, data, or biological material. |
| Recruitment | The study is strictly involving bacterial strains and their evolution and included no human participand, data, or biological material. |
| Ethics oversight | The study is strictly involving bacterial strains and their evolution and included no human participand, data, or biological material. |

Note that full information on the approval of the study protocol must also be provided in the manuscript.

# Field-specific reporting

Please select the one below that is the best fit for your research. If you are not sure, read the appropriate sections before making your selection.

☒ Life sciences ☐ Behavioural & social sciences ☐ Ecological, evolutionary & environmental sciences

For a reference copy of the document with all sections, see nature.com/documents/nr-reporting-summary-flat.pdf

# Life sciences study design

All studies must disclose on these points even when the disclosure is negative.

| | |
|---|---|
| Sample size | No statistical methods used to predetermine sample size. The number of strains used was chosen to allow for extensive downstream analysis of selected evolved populations/mutants rather than to span all available strain types of K. pneumoniae. Experimental evolution was performed with 6-10 replicate populations per strain and surface to allow for a broad sampling of evolutionary trajectories. All phenotypic experiments were repeated with at least three repetitions and the exact number of repetitions are indicated in each figure legend. |
| Data exclusions | No data was excluded. |
| Replication | Al biological and technical replicates were included in the statistical calculations and are indicated in the respective figure legend. |
| Randomization | No randomization into groups were done. |
| Blinding | No specific groupings were performed and therefore blinding is not relevant. |

# Reporting for specific materials, systems and methods

We require information from authors about some types of materials, experimental systems and methods used in many studies. Here, indicate whether each material, system or method listed is relevant to your study. If you are not sure if a list item applies to your research, read the appropriate section before selecting a response.

## Materials & experimental systems

| n/a | Involved in the study |
| --- | --- |
| ☒ | ☐ Antibodies |
| ☐ | ☒ Eukaryotic cell lines |
| ☒ | ☐ Palaeontology and archaeology |
| ☐ | ☒ Animals and other organisms |
| ☒ | ☐ Clinical data |
| ☒ | ☐ Dual use research of concern |
| ☒ | ☐ Plants |

## Methods

| n/a | Involved in the study |
| --- | --- |
| ☒ | ☐ ChIP-seq |
| ☒ | ☐ Flow cytometry |
| ☒ | ☐ MRI-based neuroimaging |

# Eukaryotic cell lines

Policy information about cell lines and Sex and Gender in Research

| | |
| --- | --- |
| Cell line source(s) | The cell lines A549 and T24 were both purchased from the ATCC (American Type Culture Collection) strain collection. |
| Authentication | None of the cell lines were authenticated further. |
| Mycoplasma contamination | None of the cell lines were controlled for Mycoplasma contamination. |
| Commonly misidentified lines (See ICLAC register) | None of the cell lines belong to commonly misidentified lines. |

# Animals and other research organisms

Policy information about studies involving animals; ARRIVE guidelines recommended for reporting animal research, and Sex and Gender in Research

| | |
| --- | --- |
| Laboratory animals | Galleria melonella larvae |
| Wild animals | The study did not include wild animals. |
| Reporting on sex | Sex was not considered in the study design since there are no reliable ways of determining sex of the moth larvae. |
| Field-collected samples | The study did not include samples collected in the field. |
| Ethics oversight | No ethics oversight or approval is nessessary for invertebrates. |

Note that full information on the approval of the study protocol must also be provided in the manuscript.

# Plants

| | |
| --- | --- |
| Seed stocks | No seed stocks or plant material was used in the study. |
| Novel plant genotypes | No seed stocks or plant material was used in the study. |
| Authentication | No seed stocks or plant material was used in the study. |
